# Structure-Based, Rational Design of T Cell Receptors

**DOI:** 10.3389/fimmu.2013.00268

**Published:** 2013-09-12

**Authors:** V. Zoete, M. Irving, M. Ferber, M. A. Cuendet, O. Michielin

**Affiliations:** ^1^Molecular Modeling Group, Swiss Institute of Bioinformatics, Lausanne, Switzerland; ^2^Lausanne Cancer Center, Lausanne, Switzerland; ^3^Department of Chemistry, New York University, New York, USA; ^4^Department of Research, University Hospital Center and University of Lausanne, Lausanne, Switzerland; ^5^Ludwig Center for Cancer Research of the University of Lausanne, Lausanne, Switzerland

**Keywords:** molecular modeling, protein-engineering, TCR, TCR-pMHC, immunotherapy, adoptive transfer, cancer

## Abstract

Adoptive cell transfer using engineered T cells is emerging as a promising treatment for metastatic melanoma. Such an approach allows one to introduce T cell receptor (TCR) modifications that, while maintaining the specificity for the targeted antigen, can enhance the binding and kinetic parameters for the interaction with peptides (p) bound to major histocompatibility complexes (MHC). Using the well-characterized 2C TCR/SIYR/H-2K(b) structure as a model system, we demonstrated that a binding free energy decomposition based on the MM-GBSA approach provides a detailed and reliable description of the TCR/pMHC interactions at the structural and thermodynamic levels. Starting from this result, we developed a new structure-based approach, to rationally design new TCR sequences, and applied it to the BC1 TCR targeting the HLA-A2 restricted NY-ESO-1_157–165_ cancer-testis epitope. Fifty-four percent of the designed sequence replacements exhibited improved pMHC binding as compared to the native TCR, with up to 150-fold increase in affinity, while preserving specificity. Genetically engineered CD8^+^ T cells expressing these modified TCRs showed an improved functional activity compared to those expressing BC1 TCR. We measured maximum levels of activities for TCRs within the upper limit of natural affinity, *K*_D_ = ∼1 − 5 μM. Beyond the affinity threshold at *K*_D_ < 1 μM we observed an attenuation in cellular function, in line with the “half-life” model of T cell activation. Our computer-aided protein-engineering approach requires the 3D-structure of the TCR-pMHC complex of interest, which can be obtained from X-ray crystallography. We have also developed a homology modeling-based approach, TCRep 3D, to obtain accurate structural models of any TCR-pMHC complexes when experimental data is not available. Since the accuracy of the models depends on the prediction of the TCR orientation over pMHC, we have complemented the approach with a simplified rigid method to predict this orientation and successfully assessed it using all non-redundant TCR-pMHC crystal structures available. These methods potentially extend the use of our TCR engineering method to entire TCR repertoires for which no X-ray structure is available. We have also performed a steered molecular dynamics study of the unbinding of the TCR-pMHC complex to get a better understanding of how TCRs interact with pMHCs. This entire rational TCR design pipeline is now being used to produce rationally optimized TCRs for adoptive cell therapies of stage IV melanoma.

## Introduction

**Figure 1:**
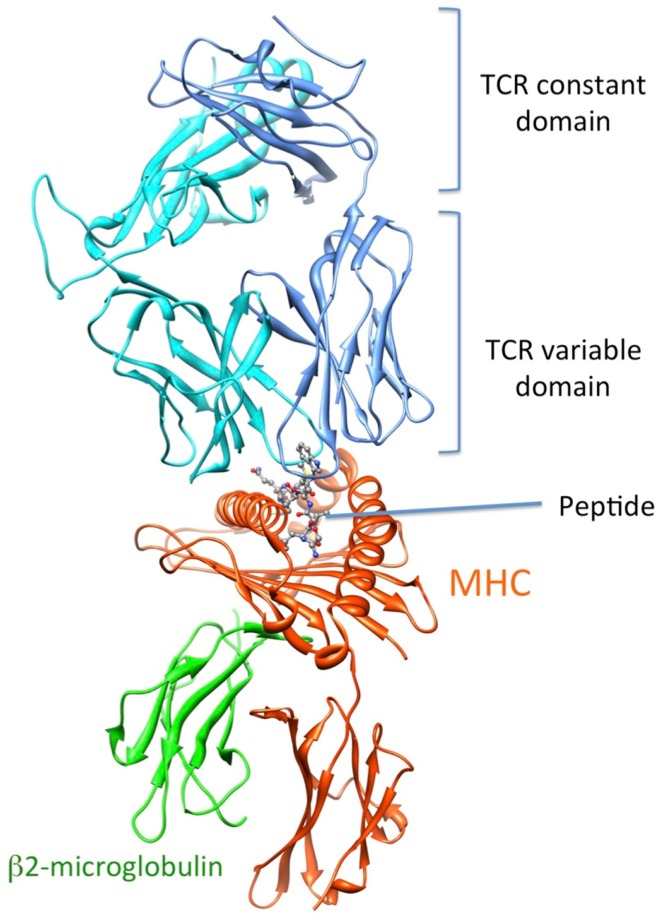
**3D-structure of the TCR-pMHC complex**. (1G4 TCR bound to NY-ESO-1/HLA-A201, entry 2BNR (124) of the PDB). TCRα, TCRβ, MHC, and b2-microglobulin are colored in blue, cyan, orange, and green, respectively. The NY-ESO-1 peptide is displayed in ball and stick.

**Figure 2:**
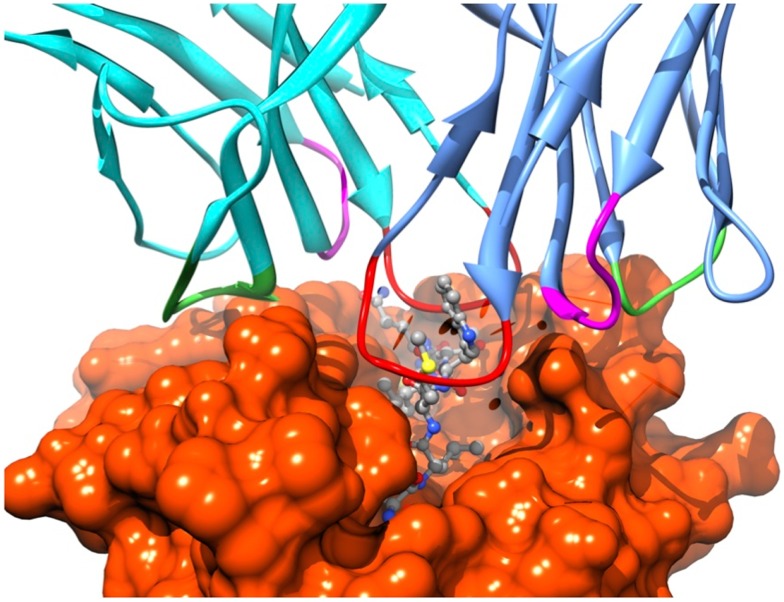
**Position of the TCR CDRs over the pMHC surface**. CDR1s, CDR2s, and CDR3s are colored in magenta, green, and red, respectively.

Recognition by the CD8^+^ T cell receptor (TCR) of immunogenic peptide (p) presented by class I major histocompatibility complexes (MHC) is a key event in the specific immune response against virus-infected cells or tumor cells. Binding of the TCR to the pMHC complex leads to T cell activation and killing of the target cell (1). The TCR is composed of two chains, α and β, that pair on the surface of the T cell to form a heterodimeric receptor on the surface of the T cell. Each chain is composed of a constant domain that anchors the protein in the cell membrane and of a variable domain that confers antigen recognition (Figure 1). The TCR contacts pMHC molecules via the 6 complementarity-determining regions (CDR), three each from the α and β chains (Figure 2). These CDRs constitute the hypervariable regions of the two V domains, called Vα and Vβ (2–4). They are generated by somatic gene rearrangement and negatively selected in the thymus against reactivity with endogenic pMHCs. CDR3α and CDR3β are the most diverse regions of the TCR and thus play a major role in antigen specificity. The CDR1 and CDR2 loops of the α and β chains predominantly make contact with the MHC molecule. The strength of the interaction between TCR and pMHC has been shown to play an important role in the T cell activation (5–9). However, the kinetics of the TCR/pMHC interaction is also determinant in T cell activation (10, 11). Consequently, understanding the biophysical properties of the TCR/pMHC interaction is of great interest for the prediction of the T cell activation, and for the rational TCR optimization toward improved adoptive transfer cancer therapy (12, 13).

This review will focus on the different computer-aided techniques we developed and used to study the TCR-pMHC complex from a structural and thermodynamic point of view. First we present the results obtained by steered molecular dynamics (SMD) simulations of molecular recognition events occurring during the TCR-pMHC complex formation. Second, we describe the different approaches we developed to derive structural models of TCR-pMHC complexes for a large TCR repertoire. Third, we summarize our approaches to estimate the binding free energy for the TCR association to pMHC. Finally, we present our *in silico* structure-based protein-engineering approach that enables the fine-tuning of TCR-pMHC binding parameters.

## Investigating TCR-pMHC Interactions Using Steered MD Simulations

The structure of ∼66 TCR-pMHC complexes are known to date (14) among which ∼25 are unique complexes. The compared features of these structures were the object of extensive reviews (1, 15, 16). In addition, binding kinetics and thermodynamics of many complexes have been measured (17). The general picture that emerges is that T cell activation requires TCR-pMHC binding to fall within a certain range of affinity and kinetics. However, clear structural determinants of TCR specificity have remained elusive. A single mutation can change a peptide from agonist to antagonist, but the same TCR can recognize various peptides with different binding modes. Thus TCRs can be exquisitely specific while displaying a high degree of cross-reactivity. In addition, TCRs use extremely varied thermodynamic strategies to bind to pMHCs, ranging from entropy-favored to entropy-opposed.

**Figure 3:**
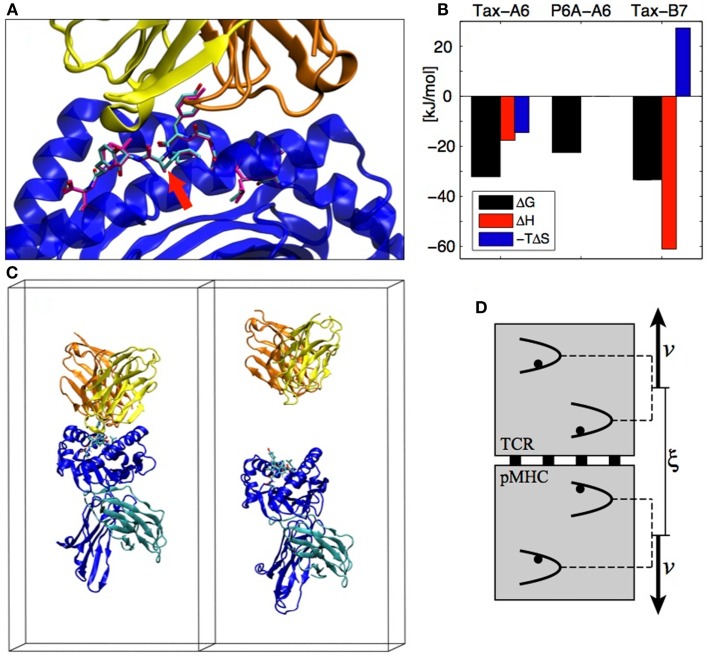
**(A)** The A6 TCR (yellow and orange) bound to the HLA-A2 MHC (blue) presenting the Tax peptide (cyan). The P6A peptide mutant is overlaid in magenta (PDB entry 1AO7). The site of the P6A mutation is indicated by a red arrow. **(B)** Experimental TCR binding thermodynamics. For P6A-A6, no entropy/enthalpy measurements are available. References are given in the text. **(C)** TCR-pMHC complex in its simulation box before and after the SMD simulation (water molecules not shown). **(D)** The individual pulling scheme used to dissociate the proteins. The distance ξ between the centers of mass is increased at rate 2*v*. Each backbone heavy atom is subjected to an individual harmonic potential. All individual potentials move in concert, with their relative positions fixed to their values in the crystal structure.

A system that exemplifies both the sensitivity of TCR recognition and its potential for cross-reactivity with different binding strategies is the Tax nonapeptide (LLFGYPVYV) from the HTLV-1 virus presented by the HLA-A0201 MHC. This pMHC is a strong agonist for the A6 TCR but the P6A peptide mutant (Pro replaced by Ala at position 6, see Figure 3A) dramatically reduces the binding affinity and abrogates T cell activation (18). On the other hand, the B7 TCR, which has the same α chain as A6 but a different β chain, is also activated by the Tax peptide presented by the same MHC. B7 binds with an affinity similar to A6, but the binding is entropically opposed, whereas A6 binding is entropically favored, outlining a completely different binding mechanism (19) (see Figure 3B).

Detailed aspects of protein–protein interactions can be characterized by SMD simulations, in which the dissociation is actuated by an external force acting on the protein. A typical reaction coordinate for protein–protein dissociation is the distance between the centers of mass of each protein. In the following, we call this reaction coordinate ξ. In the case of the TCR-pMHC, we assumed that the dissociation happens in the direction perpendicular to the cellular membranes (see Figure 3C). We also assumed that there is no substantial conformational rearrangement upon dissociation (except possibly in the CDRs and in the peptide), which is supported by the similarity of X-ray structures of bound and unbound TCRs or pMHCs. To enforce these assumptions during the SMD, we devised the individual pulling scheme (20) in which each non-H atom (except CDRs and peptide) is subjected to an individual harmonic potential. As shown on Figure 3D, the center of mass distance ξ is increased by collectively shifting the reference positions of the individual potentials. For each TCR-pMHC complex, we performed about 150 unbinding trajectories of 4 ns each during which ξ is gradually increased by 2 nm from the bound state distance to reach a final conformation as shown on the right panel of Figure 3C. The simulations were performed with the Gromacs software (21) using the Gromos 45a3 force field (22) with explicit water molecules.

**Figure 4:**
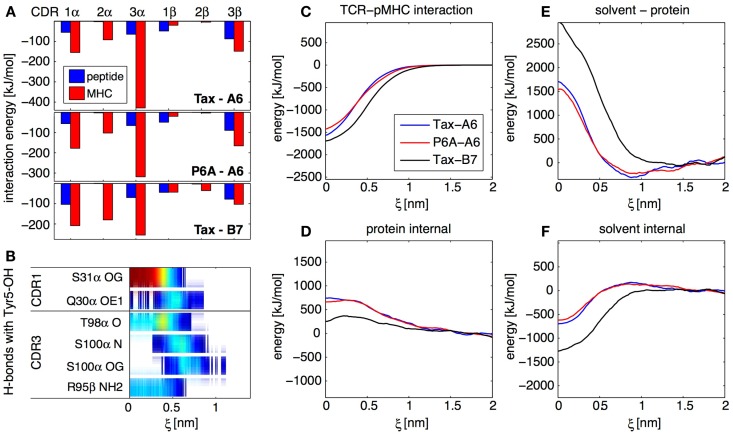
**Results from the SMD dissociation of three TCR-pMHC complexes**. **(A)** Average interaction energy in the bound complexes, partitioned in CDR and peptide/MHC contributions. **(B)** Focus on the OH group on the side-chain of the peptide Tyr 5 residue. The color bars indicate the average occurrence of H-bonds with TCR atoms as a function of center of mass separation ξ. Red means that the H-bond is formed almost all the time, blue indicate a rare interaction. **(C)** Interaction energy between the TCR and the pMHC for all three complexes, as a function of ξ. **(D)** Total internal energy (bonded and non-bonded) of TCR and pMHC. **(E)** Solvent-protein interaction energy. **(F)** Solvent internal energy. The contributions from **(C–F)** add up to the total system energy.

With about 150 trajectories we were able to obtain converged ensemble averages of many observables at any given protein–protein separation, including for highly fluctuating quantities involving solvent molecules. In particular, we established maps of H-bonds or of non-polar contacts for all residues of the TCR and pMHC as a function of ξ. As an example, Figure 4B shows an H-bond occurrence map for the OH group on the Tyr5 side-chain of the Tax peptide in the Tax-A6 system. We see that α-S31 is the main TCR H-bonding partner in the bound state, but that new H-bonds are formed with α-S100 in the transition state. Overall, our simulations have shown that the number and diversity of H-bonds occurring in a protein complex largely exceeds what is apparent from the crystal structures. Using this methodology, detailed maps such as Figure 4B can be established for any interaction to any atom in the system, depending on the biological question of interest.

For all three TCR-pMHC complexes, we monitored energy variations in different parts of the system upon dissociation. Figure 4C shows that there are differences in TCR-pMHC interaction energies between the complexes. But these are largely compensated by effects in the internal protein reorganization energy (Figure 4D), solvent-protein interaction energy (Figure 4E), and solvent internal energy (Figure 4F). As a general lesson for protein–protein interactions, we retain that the solvent plays a key role in two different ways. First, variations of solvation energies exceed contributions from the proteins themselves upon binding. Second, specific water molecules trapped at the interface can influence the binding mechanism and thermodynamics (data not shown). In the present case, these two aspects happen to be also the two major factors differentiating A6 and B7 binding.

Focusing on the bound state of the TCR-pMHC complexes, the decomposition of the average interaction energy among CDRs brings valuable insights. Figure 4A illustrates the differences between the A6 and B7 binding modes, with a much less prominent contribution of CDR3α for B7. Generally for all complexes, Figure 4A shows that, while CDR2 interacts mostly with MHC, both CDR1 and CDR3 interact equally with peptide and MHC, even at large distance, as confirmed by our H-bond occurrence maps (not shown). Therefore, our simulations do not support the two-step model (23) for TCR engagement, in which the CDR1 and CDR2 preferentially contact the MHC at large distance, while the CDR3 establishes final contacts to “read” the peptide mainly at short distances.

Overall, although the P6A-A6 complex has a very different affinity compared to the wild-type Tax-A6, both complexes share very similar features in terms of specific H-bonds or energy contributions. On the other hand, the Tax-B7 complex has a binding affinity similar to that of Tax-A6, but uses a completely different binding mechanism. The B7 TCR creates a very different set of H-bonds and hydrophobic contacts to the pMHC and makes a very different usage of the solvent, which reflects in a different partition of the binding energy. In retrospect, as noted previously by Baker and coworkers (24), it is not so surprising to observe active TCRs with very different binding mechanisms. Indeed, if TCRs are issued from random sequence variation and selection upon pMHC binding affinity and kinetics only, each TCR is likely to adopt its own unique pMHC binding strategy as long as it matches these criteria.

## TCR-pMHC Homology Modeling

### Pioneering study of TCR-pMHC homology modeling

The recent development and use of experimental techniques to determine sequences of TCRs that bind to a pMHC complex (25), led to the collection of large repertoires of TCR sequences with given pMHC specificities (26, 27). Understanding the selection mechanism that causes this gene usage can be facilitated by the introduction of structural information regarding the underlying TCR-pMHC complexes. This information can be used to identify conserved 3D binding motifs that are not obvious from repertoire sequences alone (28), to suggest explanations regarding the impact of TCR mutation on its affinity for given pMHCs (29) and ultimately to support the rational engineering of TCRs with particular binding properties (7, 30). Experimental structural techniques such as X-ray crystallography or NMR provide direct and valuable information regarding the 3D-structures of macromolecules. Unfortunately, they require the production of the protein, can be time consuming, and are thus hardly applicable to the analysis of large repertoires of tens to hundreds of TCRs. According to the 3D-structure database of the international ImMunoGenetics information system [IMGT/3D-structure-DB (31, 32)] the 3D-structure of 66 TCR-pMHC complexes have been determined experimentally so far. This number is negligible compared to the vast TCR diversity created by genetic rearrangements of the TCR V, D, and J genes. Indeed, the number of unique TCRβ chains in blood has been estimated to be of the order of 10^6^ (33, 34). There is thus a need for tools able to predict the 3D-structure of TCR-pMHC complexes from the amino acid sequences of their components.

The pioneering work of Michielin et al. (29) provided a remarkable demonstration of the feasibility and the predictive ability of TCR-pMHC 3D-structure modeling. The authors used a murine T1 TCR, specific for a photoreactive derivative of the Plasmodium berghei circumsporozoite (PbCS) 253–260 nonapeptide presented by the *K*_D_ class I MHC (35). Fifty mutants involving the TCR’s CDR, the MHC’s α1 and α2 helices and the peptide were prepared and the association constants between the TCR and the pMHC were measured (35). A first homology model was built for the wild-type TCR-pMHC complex with the MODELLER program (36–38), using the four TCR structures available at that time (39–42) and the structure of H-2Kb MHC (43) as templates. These structures provided good templates for the β-sheet framework of the TCR, and of the α helices/β-sheet of the MHC grooves. The high secondary structure content of these regions imposed strong restrictions on their backbone conformation according to the MODELLER algorithm, which facilitates the modeling of those parts. In contrast, the CDR loops of TCRs have a very low level of sequence identity and no specific secondary structure, which obviously limits the efficiency of modeling by homology. The CDR loops conformation obtained in the first homology model were thus refined using a simulated annealing technique (44), followed by clustering the generated conformations based on their relative Cartesian coordinate root mean square deviation (RMSD). A final conformation was chosen from a well-populated, low-energy cluster, whose structure was compatible with the experimental mutational data. All but three of the 50 mutations found qualitative explanation in the model in terms of breaking of a significant TCR/pMHC interaction. In addition, the model suggested that a TCR pocket could form upon binding to accommodate the peptide hapten, explaining the high level of affinity of the T1 TCR for this pMHC (*K*_D_ ∼ 10 nM), and demonstrating predictive capabilities for the modeling approach that go beyond reproducing only the structural features present in the templates. Since more X-ray structures of TCR-ligand complexes are continuously determined, it could be expected that the range of applicability and the accuracy of such a modeling approach would improve, since there is no limitation to the number of simultaneous templates that can be used.

### TCRep 3D

The first study described above (29) led to the development of TCRep 3D, as a generalization of the TCR-pMHC modeling approach (28). TCRep 3D is an approach dedicated to the prediction of high-quality 3D-structures that can provide a functional insight on the interaction between a TCR and a pMHC. It includes by design minimal input and optimal automation, to analyze wide sets of sequences of TCRs belonging to a common TCR repertoire.

**Figure 5:**
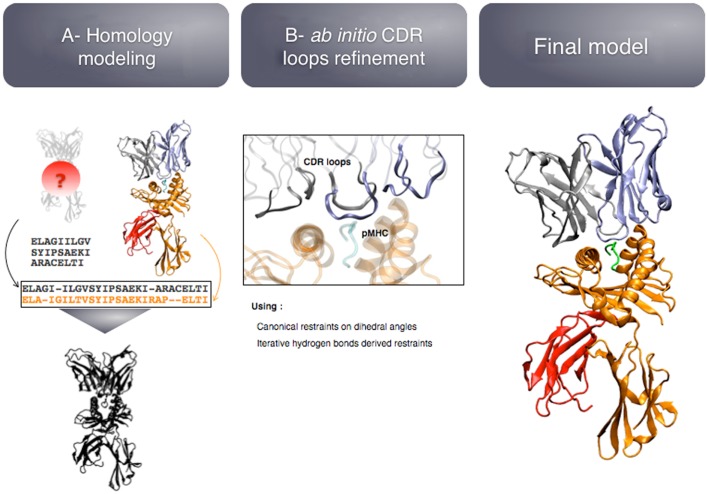
**Key steps of the TCRep 3D modeling procedure**.

The modeling pipeline is composed of two modules (see Figure 5): (i) homology modeling of the TCR-pMHC complex and (ii) *ab initio* CDR loops structure optimization. First, the user provides the sequence of the target complex and a list of preferred templates. By default, all the TCR-pMHC templates of the Protein Data Bank are used. The global structure of the complex is modeled by homology. It could be possible to couple this step to computer-aided approaches for the docking of peptide antigens into MHC molecules (45), in case the peptide binding mode could not be correctly predicted by homology modeling. Each CDR loop is then subsequently refined while the rest of the complex remains rigid. The MODELLER (38) software is used for the two modules.

The originality of TCRep 3D resides in the specific bias that we impose to the system during the structural sampling. Canonical restraints available from the literature (46) are added to the backbone dihedral angles of CDR1 and 2 to limit the conformational space accessible to the loops. We developed an iterative sampling method that identifies potential hydrogen bonds between TCR and pMHC, and converts them into modeling restraints. The scoring function was adapted accordingly, to favor structures that satisfy most of the canonical restraints, and display potential hydrogen bonds. We demonstrated that TCRep 3D is significantly more efficient than common loop modeling approaches in predicting CDR loops conformations.

At the time of the study, TCRep 3D produced one TCR-pMHC structure in 7 days on a single CPU. However, the modeling can be parallelized on a computing grid, and the computation time scales efficiently with the number of CPUs used, allowing the user to quickly model a large number of sequences.

TCRep 3D has been successfully applied to experimentally determined sets of sequences of TCRs that recognize given cancer epitopes.

**Figure 6:**
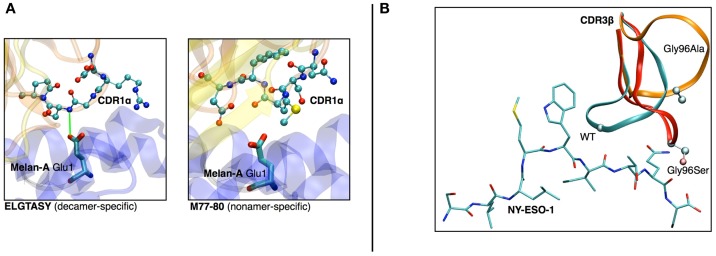
**(A)** Structural differences between nona- vs. decamer-specific TCR bound to Melan-A(ELA)/HLA− A^∗^0201. The green line identifies the hydrogen bond that is formed between CDR1α and Glu1 of the peptide. No favorable interactions were identified between M77-80 and the decamer variant of Melan-A. **(B)** *In silico* mutation results in NY-ESO-1 repertoire. The dramatic structural rearrangement of the mutated CDR3β confirms the importance of the central Gly.

(a) In a study on HLA-A^∗^0201/Melan-A-specific CD8 T cells (47), the modeling of the TCR-pMHC 3D-structure revealed the structural feature that explained how two distinct sets of TCR performed differently in recognizing a naturally occurring decamer variant of the Melan-A peptide. One of the TCR subsets could not make proper interactions with the glutamic acid at position 1 of the peptide because of the location and structural properties of the CDR1α (see Figures 6A,B).
(b) The analysis of HLA-A^∗^0201/NY-ESO-1_157–165_ specific CD8^+^ T cells from five melanoma patients showed a preferential usage of three Vβ genes. Additionally, experimental evidence on the importance of the Met4-Trp5 pair of the NY-ESO-1_157–165_ antigen were found, suggesting that those two contact residues make critical interactions with the TCR, regardless of the gene segment usage (26). The modeling of the corresponding TCR-pMHC structures revealed a striking mechanism of selection through the presence of a single conserved glycine residue situated in the center of all CDR3β. An *in vitro* experimental functional study of mutations of this amino acid combined with *in silico* modeling of several mutants was performed. All mutations resulted in dramatic structural changes associated with complete experimental loss of affinity of the TCR to NY-ESO-1/HLA-A^∗^0201 (28).

### Long-range driving force for TCR orientation

Over the years, successive releases of TCR-pMHC crystal structures have revealed the variety of native binding orientations that the TCR can adopt. Recent studies reported a range of more than 45° in the TCR binding angles relative to the MHC (48), depending on the peptide, the MHC, and the α/β pairing of the TCR. Although the challenge of TCR binding mode prediction has been recurrently discussed, only a few studies have focused on predicting the actual binding mode of given TCR-pMHC (49, 50). Therefore, all methods and applications relied on the existence of at least one TCR-pMHC crystal structure.

**Figure 7:**
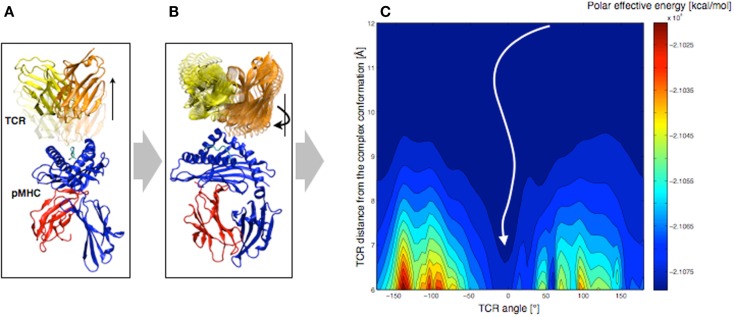
**Rigid displacement protocol**. **(A)** Rigid TCR translation along the principal axis. **(B)** Rigid TCR rotation around the principal axis. **(C)** Landscape representation of the TCR-pMHC polar energy as a function of the TCR/pMHC distance and the TCR angle. 0° corresponds to the native orientation of the bound conformation.

In order to understand the molecular basis that governs TCR orientation upon binding, we tested a simplified rigid approach on all published TCR-pMHC crystal structures (48), which allowed scanning quickly multiple orientations of the TCR relative to the pMHC. In this approach, the TCR was moved 6–12 Å away from the pMHC molecule along the TCR principal axis (see Figure 7A). Subsequently, the TCR was rotated around that same axis until a complete revolution was obtained (see Figure 7B). The effective energy of the system was computed every 5°, as the sum of the intermolecular energy and the solvation free energy, using the CHARMM22 force field (51, 52) in combination with the FACTS implicit solvation model (53).

We demonstrated that the sum of the Coulomb interaction and the electrostatic solvation energies is sufficient to identify the native TCR orientation as the energetic minimum upon rotation (see Figure 7C). Importantly, despite the rigid-body simplification, the results were robust upon small structural variations of the TCR such as changes induced by MD simulations. We also tested our approach on crystal structures of unbound TCRs, which were confronted to pMHCs. Accurate energy minima were also identified, suggesting that perfect shape complementarity is not required to obtain a reliable signal. The long-distance interactions during the TCR approach appear to be independent of the binding process itself, since the binding orientation is reliably identified without considering either short-range energy terms or CDR induced fit upon binding.

Furthermore, we decomposed the effective energy into per-residue contributions, in an approach that is similar to the Molecular Mechanics – Generalized Born Surface Area (MM-GBSA) energy decompositions (54). The contributions of structural sub-groups to the profile of the TCR/pMHC interaction energy during rotation were calculated, to estimate their role in the overall orientation. Results showed that most of the driving force (>90%) leading to the orientation of the TCR is defined by CDR1,2/MHC interactions. This is in agreement with previous observations, revealing a ring of charged residues at the pMHC interface, which interacts with CDR1,2 with complementary charges (48). We reported that the role of the CDR3/peptide interaction is of lesser importance at long-distance.

In turn, such knowledge of the structure may be used as a preliminary approach in the process of modeling protein/protein interactions. More specifically, the rigid search for an energetic minimum upon TCR rotation may become a complementary module of TCRep 3D, to search for the correct binding mode, after modeling the TCR and the pMHC independently. We attempted to predict the binding mode of the A6 TCR with tax/HLA-A^∗^0201, after modeling the TCR by homology. The effective energy minimum upon rotation was computed for 500 homology models, and we obtained an average shift of 12.2° from the orientation of the crystal structure. This demonstrated the potential of the approach as a component of a TCR-pMHC structural prediction pipeline (55). The approach is also easily applicable to other types of protein complexes, provided that the association is also driven by long-range electrostatic interactions.

## Free Energy Calculations

### Application of the thermodynamic integration method for TCR-pMHC binding free energy differences

T cell receptor recognition can exhibit exquisite specificity upon single peptide mutation. In the A6/Tax/HLA-A0201 complex described above (Tax-A6), mutating the Ala at position 6 to a Pro (P6A) turns the Tax peptide from a strong agonist into a weak antagonist. These systems were extensively studied experimentally (18, 56) and the binding free energy difference between the Tax-A6 and the P6A-A6 complexes was found to be ΔΔ*G* = 2.90 ± 0.20 kcal/mol. (see Figure 3B). These results are difficult to rationalize from the structure alone, as there is almost no difference between the conformations of the Tax-A6 and P6A-A6 complexes (see Figure 3A, red arrow). To gain a better understanding of the effect of the mutation on TCR recognition, we used free energy simulation to analyze in detail the origin of the binding free energy difference (57).

**Figure 8:**
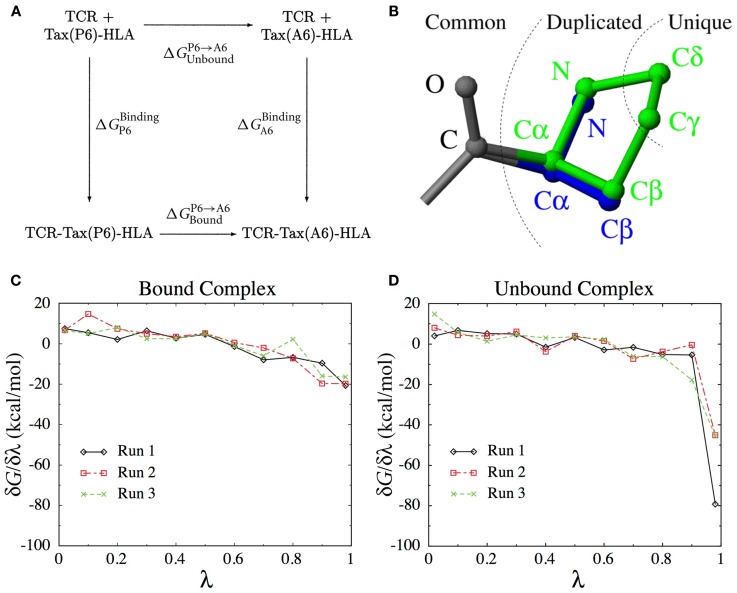
**(A)** The thermodynamic cycle underlying Eq. (1). **(B)** The dual topology scheme used to interpolate the potential energy function between the Pro side-chain (green) and the Ala side-chain (blue). Common atoms are unaffected, Duplicated atoms change type and non-bonded parameters. Unique atoms vanish, with their non-bonded interactions switched to zero and their bonded interactions unchanged. **(C)** Derivatives of the free energy obtained for each λ-value in three independent runs for the bound complex. **(D)** Idem for the unbound complex.

As we will see below, calculating the binding free energy by simulating the entire TCR-pMHC unbinding process itself is difficult. Instead, the present method uses the thermodynamic cycle shown in Figure 8A to reformulate the problem,

(1) $$\Delta \Delta G_{\text{P6A}}^{\text{Binding}}=\Delta G_{\text{P6}}^{\text{Binding}}-\Delta G_{\text{A6}}^{\text{Binding}}=\Delta G_{\text{Bound}}^{\text{P6}\to \text{A6}}-\Delta G_{\text{Unbound}}^{\text{P6}\to \text{A6}}.$$

This means that we can obtain $\Delta \Delta G_{\text{P6A}}^{\text{Binding}}$ by computing the P6 → A6 mutation free energy in both the unbound and the bound states. Among the different methods available to calculate mutation free energy differences (58, 59), we chose thermodynamic integration (60, 61). We define an interpolated potential energy function *U*(*r*, λ) that is equivalent to the potential energy function of the wild-type for λ = 0 (Pro) and to that of the P6A mutant for λ = 1 (Ala). The free energy difference can be obtained through

$$\Delta G_{\text{Bound}}^{\text{P6}\to \text{A6}}=\int_{0}^{1}{\left\langle \frac{\mathrm{\partial U}\left(r,\lambda \right)}{\partial \lambda }\right\rangle }_{\lambda }d\lambda .$$

Here, ${\left\langle \cdot \right\rangle }_{\lambda }$ represents an ensemble average at fixed λ. In practice, we perform a set of simulations at discrete λ-values and evaluate the integral above numerically. The number and position of λ-values required for accurate integration depends on the smoothness of the ${\left\langle \frac{\mathrm{\partial U}}{\partial \lambda }\right\rangle }_{\lambda }$ function. To construct an appropriate interpolating potential energy function, a dual topology scheme was used, as shown on Figure 8B. For vanishing atoms, only the non-bonded interactions are scaled, while the bonded interactions are left unchanged (62). As we did not use soft-core potentials (63) for vanishing atoms, special care was taken in the limit of λ → 0 and λ → 1 to deal with the singularities of the Coulomb and Lennard-Jones potentials.

Starting from the crystal structures for both the unbound (64) and the bound complex (18), MD simulations were performed in the CHARMM program (51) with the CHARMM22 force field (52). The proteins were locally solvated in a sphere of 16 Å surrounding the peptide using the stochastic boundary method (65). The set of λ-values used were λ = 0.02, 0.1, 0.2, 0.3, 0.4, 0.5, 0.6, 0.7, 0.8, 0.9, 0.98. After 100 ps of initial equilibration, data was collected for 30 ps at each λ-value, separated by 10 ps equilibration time after each λ update. Three simulations with independent initial velocities were produced.

The simulations were structurally stable with average RMSD of non-H atoms no greater than 0.8 Å with respect to the crystal structures. The average free energy derivatives obtained in the three different runs are shown on Figures 8C,D for the bound and unbound states, respectively. In the unbound state (Figure 8D), the derivative takes very large values close to λ = 1, due to the interaction of the vanishing Pro atoms with the solvent molecules. This does not happen in the bound state, because the vanishing atoms are concealed from the solvent in the void created by the protein pocket.

Three different schemes were tested for the extrapolation to λ = 0 and λ = 1, using linear, quadratic or λ^−3/4^ functions. The integration over all λ-values was performed using the trapezoidal rule. The final result is $\Delta \Delta G_{\text{P6A}}^{\text{Binding}}=2.9\pm 1.1\;\text{kcal/mol}$, which compares very favorably with the experimental value of 2.9 ± 0.2 kcal/mol.

One of the major strengths of the method lies in the linearity of Eq. (1), which allows decomposing as a sum of contributions form different types of interactions and/or of different parts of the system. Given that the total free energy difference is in good agreement with experiment, there is a good chance that the decomposition provides meaningful insights on the mechanisms leading to TCR specificity.

A notable contribution to ΔΔ*G* (+0.64 kcal/mol) arises from the difference in solvation free energy of the mutated residue: in the unbound structure, the Tax P6 residue is solvent-exposed with around 35% of its surface accessible to water molecules. The A6 mutant has fewer exposed hydrophobic groups, which entails a more modest solvation penalty in the unbound state. This stabilizes the unbound state of the P6A mutant relative to the wild-type, which in turn makes P6A-A6 binding less favorable The rest of the peptide contributes a modest 0.38 kcal/mol to ΔΔ*G*.

The total contribution of the TCR in the bound state is around +0.8 kcal/mol destabilizing the P6A mutant, the most significant part of which is due to the CDR3α loop. Most of this energy arises from the van der Waals term, in accord with the fact that the TCR provides good surface complementarity for the hydrophobic side-chain of the Pro residue. Since the pocket is already present in the Tax-A6 complex, there is no large free energy cost needed to induce it, in contrast to what is found in the solvent. The TCR residues that contribute the most to the TCR specificity for the wild-type peptide are N30 from the CDR1α, D99, and S100 from the CDR3α, and G97, L98, A99, G100, G101 from the CDR3β.

The most important contribution to ΔΔ*G* (1.26 kcal/mol) arises from the difference in interactions with the MHC. This is due to a conformational change that takes place in the Tax P6 region upon TCR engagement. In the Tax system, the cost of this conformational change is balanced by a very favorable interaction of the Pro ring with hydrophobic residues of the MHC groove, which does not take place with the shorter Ala side-chain. This is an example of how a conformational change taking place along the physical binding pathways translates into a free energy contribution along the alchemical pathways.

Overall, it emerges that the total binding free energy difference between the wild-type and the mutant peptide consists of four contributions that are similar in magnitude. The self-interaction of the peptide and the change in the interaction between the peptide and the three portions of its environment (TCR, HLA-A2, and solvent) all contribute between 0.5 and 1.2 kcal/mol to stabilizing the wild-type complex. This important result was not evident from the X-ray structures or the experimental data. Interestingly, these calculations show that accurate free energy differences could be obtained although most of the complexity of this system was ignored in our relatively short simulations including only the mutated side-chain and its local environment.

### Assessing the applicability of the Jarzynski identity to calculate TCR-pMHC binding free energy profiles

In classical thermodynamics, the dissipative work W_A → B_ needed to bring a system from state A to state B is greater than the free energy difference Δ*G*_AB_ between the two states, with equality only in adiabatic conditions. Conversely, a recent result in non-equilibrium statistical mechanics, the Jarzynski identity (JI) states that (66, 67)

$$e^{-\beta \Delta G_{\text{AB}}}={\left\langle e^{-\beta W_{A\to B}}\right\rangle }_{0}.$$

Here, $\beta ={\left(k_{\text{B}}T\right)}^{-1}$, with *T* the temperature and *k*_B_ the Boltzmann constant. The average ${\left\langle \cdot \right\rangle }_{0}$ is taken over canonically distributed initial conditions in state *A*. The JI was proven to hold in the case of thermostated molecular dynamics (66–69). The JI was applied with some success to simulations of small molecular systems (68–71). Given the biological importance of the TCR-pMHC system, we were compelled to determine if the JI could be employed to calculate protein–protein binding free energy profiles from SMD trajectories of large protein–protein complexes (72). The free energy profile, or potential of mean force (PMF), is very relevant because in addition to the total binding free energy it provides estimates of *k*_on_ and *k*_off_ from the free energy barrier height.

In SMD, an external potential of the form u(ξ(*r*), *t*) is used to steer the system from position *A* at time 0 to *B* at time *t* along the reaction coordinate ξ. This potential can be a simple harmonic potential or take a more complex form such as in the individual pulling scheme (72) presented in Section “Investigating TCR-pMHC Interactions Using Steered MD Simulations.” If we are interested in the PMF along ξ, the JI can be written as (73),

$$e^{-\beta G\left(\xi \right)}\alpha {\left\langle \delta \left[\xi \left(r\right)-\xi \right]e^{-\beta W\left(t\right)}\right\rangle }_{0}.$$

Importantly, *W*(*t*) is the work accumulated by the perturbed system (including *u*), defined as (74),

$$W\left(t\right)=\int_{0}^{t}dt\frac{\partial u}{\partial t}\left(\xi \left(r\right),t\right).$$

**Figure 9:**
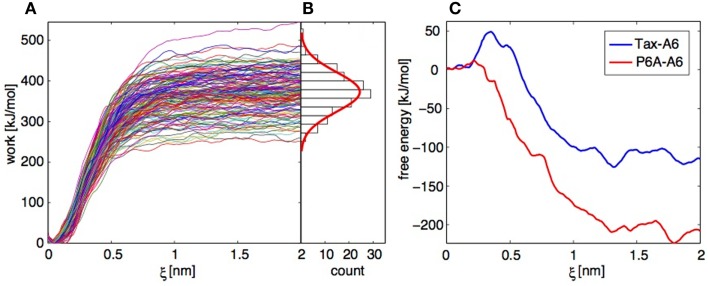
**Application of the Jarzynski identity to TCR-pMHC dissociation**. **(A)** Work profiles collected in 152 independent trajectories for the Tax-P6A complex. **(B)** Histogram of the work distribution at ξ = *2* nm, with a fitted Gaussian distribution. **(C)** Free energy profiles calculated using the Jarzynski identity and the cumulant expansion method.

For each of the Tax-A6 and P6A-A6 complexes, we performed 150 trajectories starting from independent conformation of the bound complex. The TCR and pMHC centers of mass separation was increased by 2 nm over 4 ns. The resulting work profiles are shown on Figure 9A and the distribution of final work values is shown on Figure 9B. As expected if the system is not too far from equilibrium (71, 75), the distribution (in its central part) close to a Gaussian. To obtain the PMF, the work profiles *W*(*t*) collected from multiple SMD simulations have to be postprocessed with three distinct operations:

1. Reduce from the biased system to the physical system (unbias);
2. Average over all ξ(*r*) visited during the evolution to recover G(ξ);
3. Estimate the exponential average ${\left\langle e^{-\beta W}\right\rangle }_{0}$.

Operations 1 and 2 are performed within a modified weighted histogram scheme (73) adapted to the case of the individual potentials (72). Operation 3 can be more problematic. If we apply direct exponential averaging, the estimated PMF is very close to the lowest measured work profile at a given ξ. In the case where the true free energy value lies in the unsampled lower tail of the work distribution, direct averaging will result in a large overestimation. Indeed, for the Tax-A6 complex, the work profiles of Figure 9A would result in a dissociation free energy around 250 kJ/mol, compared to the experimental value of 32.2 kJ/mol (18).

Instead of direct averaging, if we assume that the work distribution is Gaussian, we can estimate *G*(ξ) with a second-order cumulant expansion (71, 75),

$$G\left(\xi \right)=\bar{W}\left(\xi \right)-\frac{\beta }{\text{2}}\sigma^{\text{2}}\left(\xi \right).$$

Here, $\bar{W}\left(\xi \right)$ is the mean and σ the standard deviation of the work values at ξ obtained by applying operations 1 and 2 above to each trajectory independently. The resulting PMFs are shown on Figure 9C. The final dissociation free energies are −110 and −210 kJ/mol for Tax-A6 and P6A-A6, respectively, which is a severe underestimation of the experimental values of 32.5 and 22.5 kJ/mol. We note that the calculated values should be corrected for translational and rotational entropic contributions to be compared to standard state free energy measurements, but this does not improve the results.

Our results show that direct averaging produces a strong overestimation of *G*(ξ), which could be fixed only by sampling an extremely large number of trajectories to get enough low work values. Conversely, the cumulant expansion produces a strong underestimation of *G*(ξ), which shows that the real work distribution has a shorter lower tail than the Gaussian distribution. Repeating the calculations with datasets of similar sizes and slightly different conditions showed the reproducibility of these findings.

Overall, this example illustrates the severe sampling difficulties that hamper the application of the JI to systems with sizeable dissipation. These difficulties have been evidenced by other researchers in systematic convergence studies of the JI method (76–79).

### Estimation of residue contribution to the binding free energy of the TCR/pMHC association using MM-PB(GB)SA

In the Molecular Mechanics – Poisson Boltzman Surface Area (MM-PBSA), or its variant the MM-GBSA, the binding free energy, Δ*G*_bind_, is written as the sum of the gas phase contribution, $\Delta H_{\text{bind}}^{\text{gas}}$, the desolvation free energy of the system upon binding, $\Delta G_{\text{desolv}}$, and an entropic contribution, −TΔS (80):

$$\Delta G_{\text{bind}}=\Delta H_{\text{bind}}^{\text{gas}}+\Delta G_{\text{desolv}}-T\Delta S.$$

The term $\Delta H_{\text{bind}}^{\text{gas}}$ is constituted by the van der Waals (Δ*H*_vdw_) and electrostatic (Δ*H*_elec_) interaction energies between the two partners in the complex, and their conformational energy change upon binding, Δ*H*_intra_:

$$\Delta H_{\text{bind}}^{\text{gas}}=\Delta H_{\text{elec}}+\Delta G_{\text{vdW}}+\Delta H_{\text{intra}}.$$

Δ*G*_desolv_ is the difference between the solvation free energy, Δ*G*_solv_, of the complex and that of the isolated parts. Δ*G*_solv_ is divided into the electrostatic, Δ*G*_elec, solv_, and the non-polar, Δ*G*_np, solv_, contributions,

$$\Delta G_{\text{solv}}=\Delta G_{\text{elec,solv}}+\Delta G_{\text{np,solv}}.$$

Δ*G*_elec, solv_ is calculated by solving the exact Poisson or the Poisson–Boltzmann (PB) equation (81, 82) in MM-PBSA, and the much faster but approximate Generalized Born (GB) model (83) in MM-GBSA (84). The term Δ*G*_np,solv_, which can be considered as the sum of a cavity term and a solute–solvent van der Waals term, is assumed to be proportional to the solvent accessible surface area (SASA) (85),

$$\Delta G_{\text{np,solv}}=\gamma SASA+b.$$

The entropy term can be decomposed into translational, *S*_trans_, rotational, *S*_rot_, and vibrational *S*_vib_, contributions. These terms are calculated using standard equations of statistical mechanics (54, 86).

In the standard MM-PB(GB)SA protocol, all these energy terms are typically averaged over several hundreds of frames extracted from multi-nanosecond MD simulation trajectories, generally performed in explicit solvent. Those explicit water molecules are removed prior to energy calculations. In principle, three trajectories should be performed, one for the complex and one for each of the isolated partners, and the energy terms calculated using the corresponding simulation. However, a frequent, less computationally demanding, approximation consists in performing only one MD simulation for the complex (54). The terms relative to one isolated partner are then calculated after removing the atoms of the other partner in the frames extracted from the MD simulation of the complex. In this variant, the conformational energy change upon association is therefore neglected (Δ*H*_intra_ = 0), and the influence of conformational changes on the other energy terms are not captured.

MM-PB(GB)SA has been used successfully to identify the hot-spots of protein–protein association and to determine the effect of mutations on association processes (84, 87–90). Two approaches can be considered. The computational alanine scanning (CAS) approach (91) is directly comparable to its experimental counterpart. In this approach, Δ*G*_bind_ values are calculated for the wild-type system, as well as for several mutants in which one residue has been replaced by an alanine. The difference in Δ*G*_bind_ between the wild-type system and the mutants can be used to estimate the role played by each residue in the association process. Alternatively, it is possible to perform a binding free energy decomposition (BFED) for the wild-type system (84). In this approach, the contributions to Δ*G*_bind_ arising from groups of atoms, typically single residues, or even backbone or side-chain, are estimated from the wild-type system by performing a pairwise decomposition of the MM-PB(GB)SA terms (88, 90). BFED, which requires only one binding free energy calculation, is faster than CAS. In addition, BFED provides the possibility to study contributions from non-mutable groups of atoms, such as the backbone. However, contrarily to CAS, BFED results cannot be compared directly to an experimental alanine scanning.

To assess the ability of the MM-GBSA approach to identify quantitatively the hot-spots residues for the TCR/pMHC association, we performed a study of the 2C TCR/SIYR/H-2Kb system using both the CAS and BFED methods (90). This system was chosen because both the experimental 3D-structure and the results of an experimental alanine scanning were available at that time (92). A very good correlation was found between the residue contributions to Δ*G*_bind_ from both methods, with a correlation coefficient of 0.94, highlighting the interest in the faster BFED approach. A correlation coefficient of *R* = 0.67 was found between experimentally determined activity differences for alanine mutants and the calculated binding free energy changes upon mutation.

Our results also showed that BFED provided a more detailed and reliable description of the interactions between the TCR and pMHC molecules when including entropic terms. When the entropy was taken into account, the correlation coefficient was increased to 0.72. It was noticeable that the correlation obtained when neglecting the entropy term, which is very computationally expensive to calculate, was sufficient to quantify and rank the importance of the residues for TCR/pMHC association. Altogether, these pioneering results suggested that the BFED for the TCR-pMHC system provides a detailed and reliable enough description of the interactions between the molecules to be used as an *in silico* investigation tool in TCR protein-engineering.

## Computer-Aided Protein Engineering

### Background

Patients with diverse types of cancer develop tumor-specific CD4^+^ and CD8^+^ T cell responses. Although these responses are typically unable to contain solid tumor growth or hematological malignancies, clinical studies have revealed the adoptive transfer of *ex vivo* expanded autologous tumor-specific T cells to be a promising immunotherapeutic approach to cancer treatment (13). A limitation, however, is that TCRs which bind tumor associated/self antigen are often of relatively low affinity. TCRs generally bind pMHC in the range of *K*_D_ = 1–100 μM (93). However, as a result of the thymic negative selection whereby T cells with high-affinity TCRs for “self” antigens are eliminated to prevent autoimmunity, TCRs specific for “self” tumor associated antigens tend to be weaker-binders compared to TCRs specific for “non-self” peptides (94). Thus, the development of tumor-targeting TCRs to endow them with optimal binding properties, both in terms of fine-specificity against the targeted pMHC, and kinetic/affinity parameters that confer maximum cellular responsiveness, is a field of intense research toward cancer immunotherapy development (95).

The relative importance of the roles played by the TCR/pMHC binding affinity (*K*_D_), and individual kinetic parameters (*k*_on_ and *k*_off_), on T cell activation, has been intensively studied recently (11). The emerging consensus hypothesizes the existence of a TCR/pMHC “dwell-time”(96) enabling the sequential interaction of TCRs with a rare antigenic pMHC complex – a process known as “serial triggering” – and conferring an optimal T cell activation (97). It has also been demonstrated that both *k*_on_ and *k*_off_ define the “effective half-life” of a TCR/pMHC interaction (98). Thus, *K*_D_, *k*_on_, and *k*_off_ do all contribute to T cell activation and their optimizations should be addressed concomitantly by TCR engineering techniques for cancer immunotherapy.

As introduced earlier, TCRs contact pMHC antigens via the six CDRs (Figure 2), with CDR3α and CDR3β mainly bound over the peptide, and CDR1 and CDR2, α and β, making more contacts with MHC. It could thus be expected that mutations of CDR3s would be more likely to maintain peptide specificity than mutants of CDR1s and CDR2s. Indeed, several mutagenesis studies produced high-affinity TCRs bearing mutations on CDR3α and CDR3β that were found to be peptide-specific (99–102). However, other studies also discovered high-affinity mutants in CDR1 and CDR2 retaining peptide specificity despite the close proximity of the mutated region to MHC residues (99, 103). This indicates that all six CDRs can serve as a focus for mutagenesis to generate higher-affinity TCRs, while still potentially retaining substantial peptide specificity (2, 93).

Several efforts have been performed to optimize TCRs (93), which mainly consist in experimental yeast (100, 101, 104–106), phage (18, 102, 103, 107), and mammalian cell (108, 109) display techniques. These approaches were able to increase the affinity of the TCR by a factor of 100–10^6^, leading to *K*_D_ as low as 26 pM (102). Although very efficient to increase the *K*_D_, these techniques lead to TCRs bearing multiple mutations, without providing a straightforward control of the effect of each one. Such TCRs are prone to alloreactivity due to peptide-independent binding of MHCs (95, 110).

Detailed control of the effect of each mutation at the atomic level can be provided by *in silico* rational protein-engineering techniques (111–115). Recently, Haidar et al. (116) engineered the human A6 TCR for enhanced affinity toward the Tax peptide/HLA-A2 MHC complex. Rapidly, the authors created a set of 219 fitted scoring functions, aiming to reproduce the binding affinity change upon 648 mutations of the ovomucoid turkey inhibitor molecule, using energy and statistically derived potential terms. Each function was then tested against the affinity changes of a first set of 11 A6 TCR mutants, and evaluated by correlation. The function reproducing the best the affinity changes on A6 TCR (named ZAFFI score) was retained to suggest new mutations. Due to the significant number of non-binding mutations generated using only the ZAFFI score, the authors further developed the ZAFFI filter function. The latter, trained by a Monte Carlo method on the 36 first A6 TCR point mutations, was employed to filter out mutations with potentially bad electrostatic contacts. In total 59 mutants were tested. Twelve were found to be better binders than the wild-type TCR, as measured experimentally by Surface Plasmon resonance (SPR). It must be noted that some non-binders were generated on purpose to help training the ZAFFI score and filter. All mutations found in this study to increase the binding were hydrophobic substitutions that enhanced the interface complementarity. No mutation introducing new significant electrostatic contacts, and thus potentially increasing the selectivity of the TCR/pMHC binding, was found positive. Despite the use of fitted scoring function and filter that hampers its straightforward translation to non-TCR systems, this interesting study illustrated the feasibility of a rational *in silico* approach to design TCR with higher affinities. It opened the road to new approaches, with physically sound and non-fitted universal free energy estimates, straightforward transferability, and high success rates.

### Methodology and results on BC1 TCR

Encouraged by the results of our BFED method in reproducing the outcome of a alanine scanning experiment on the TCR/SIYR/H-2Kb system (90), we decided to develop a new structure-based approach, based on MM-GBSA free energy calculations, to rationally design new TCR sequences. Our approach can be divided into several steps. First, the importance of each wild-type TCR residue for the TCR/pMHC association is estimated using a MM-GBSA BFED. Then, based on the TCR-pMHC structure and the residue contributions to the binding, mutations are designed for the residues showing the most promising opportunities of enhancement for the interaction with the pMHC. These putative sequence modifications are finally selected for experimental testing based on the estimated binding free energy gain, ΔΔ*G*_bind_. The latter was obtained by calculating the contribution of each residue to the binding free energy change upon a given mutation, $\Delta \Delta G_{\text{bind}}^{\text{res}}$, as the difference between the residue contribution for the mutated complex, $\Delta G_{\text{bind}}^{\text{res,mut}}$, and that for the wild-type complex, $\Delta G_{\text{bind}}^{\text{res}}$:

$$\Delta \Delta G_{\text{bind}}^{\text{res}}=\Delta G_{\text{bind}}^{\text{res,mut}}-\Delta G_{\text{bind}}^{\text{res}}.$$

$\Delta G_{\text{bind}}^{\text{res}}$ and $\Delta G_{\text{bind}}^{\text{res,mut}}$ values were calculated from MD simulations of the wild-type and the corresponding mutated TCR-pMHC, respectively. The binding free energy difference upon a given mutation, ΔΔ*G*_bind_, was finally obtained by summing the $\Delta \Delta G_{\text{bind}}^{\text{res}}$ values over the mutated residue and all the residues in contact with it. This local summation was preferred to a summation over all residues of the TCR-pMHC complex since it suppresses the errors arising from residues far from the site of the sequence modification, and making no contribution to the mutation effect.

We have applied our computer-aided protein-engineering approach to the BC1 TCR (26, 117). This TCR was discovered from a long-surviving melanoma patient (LAU #155) with a naturally occurring CD8^+^ T cell response against the immunodominant cancer-testis epitope NY-ESO-1_157–165_ (SLLMWITQC), presented by the commonly expressed MHC class I allele HLA-A^∗^0201. The expression profile of NY-ESO-1 makes it an attractive target for cancer immunotherapy. It is indeed expressed by several solid tumors, including melanoma, as well as hematological malignancies (myelomas, lymphomas, and leukemias), while in normal tissue its expression is limited to the testis cells (118–122). Interestingly, recent studies have shown that an immune response against NY-ESO-1 can convey an important clinical benefit for the patient. Seventy-seven percent of patients treated with the CTLA-4 blocking antibody ipilimumab showed favorable clinical outcome if they had a detectable CD8^+^ T cell responses against NY-ESO-1, compared to only 14% otherwise (123). Our *in silico* approach was facilitated by the existence of the crystal structure of the 1G4 TCR bound to NY-ESO-1/HLA-A^∗^201 (124), PDB ID 2BNR, available in the protein databank (125). The sequence of the latter differs only from that of the BC1 TCR by four residues: Thr95α, Ser96α, Asn97β, and Thr98β of the 1G4 TCR, are replaced by Gln95α, Thr96 α, Ala97β, and Ala98β in BC1 TCR, respectively. These sequence modifications were introduced in the 1G4 X-ray structure before applying our approach. Noticeably, three crystal structures of free and bound 1G4 TCR were solved after we performed our simulations (126). These affinity-enhanced TCRs contain mutations in the CDR3 loops or in both the CDR2 and CDR3 loops, which were obtained by *in vitro* directed evolution (102). They revealed that the binding mode for the high-affinity TCRs was identical to that of the wild-type TCR, with only limited changes in the mutated CDRs. A previous assessment by Zhao et al., of six phage library-derived 1G4 TCR variants demonstrated three categories of TCR specificity related to affinity; (i) super-high-affinity TCR (26 pM) which completely lacked specificity, (ii), mid-range affinity TCR (5 and 85 nM) that were specific only in the absence of CD8 co-engagement, and, (iii), intermediate-range affinity TCR (0.4 and 4 μM) that maintained specificity (107). By taking a rational computer-aided approach to TCR engineering, we were able to design a new original set of TCR variants. We enhanced the TCR/pMHC binding interactions in a “step-by-step” manner, targeting change to specific kinetic parameters, and limiting overall gain in affinity as well as potential for cross-reactivity.

**Figure 10:**
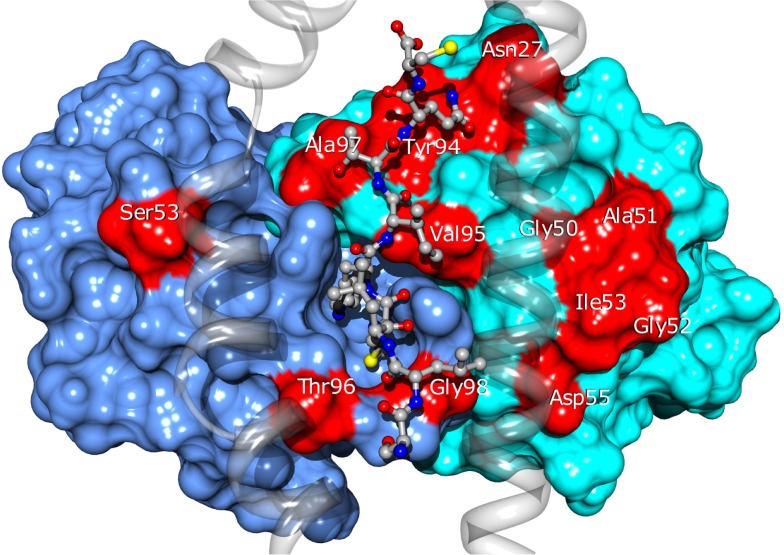
**Location of the BC1 TCR residues for which sequence modifications were designed**. TCRα and β are shown as blue and cyan surfaces. Modified residues are colored in red. The NY-ESO-1 peptide is shown in ball and stick and the MHC as a transparent ribbon. Only the α1 and α2 helices of MHC are displayed, for the clarity of the figure.

Twenty four of the most promising mutations identified using this approach, spanning 11 different residues of the CDRs (see Figure 10), were introduced into the BC1 TCR sequence. The engineered proteins were produced using a mammalian cell expression system, purified and tested by titration ELISA. We found a qualitative agreement between the calculated ΔΔ*G*_bind_ values and the experimental results. Thirteen (54%) of the mutations proposed by our approach showed improved affinity for the pMHC, compared to the wild-type TCR (Zoete et al., in preparation). We obtained an excellent correlation of *R* = 0.85 between the calculated ΔΔ*G*_bind_ and the measured co-logarithm of the optical density measured by ELISA titration. Only three outliers were found: Vβ A51V, Y94N, and A51D. Their presence might be explained by conformational rearrangements upon mutation, minimally accessible during the MD simulations. This correlation illustrates the efficiency with which the ΔΔ*G*_bind_ calculated with our method allows for the rational selection of TCR sequence modifications potentially increasing its affinity for pMHC.

For quantitative TCR/pMHC binding measurements some of the TCRs were refolded from bacterial inclusion bodies and analyzed by SPR. We began by producing TCR having one amino acid change. The best single replacement TCR had 52-fold improvement in *K*_D_ (Zoete et al., in preparation). Then, we gradually increased affinity by step-wise combination of rationally selected replacements in both the β-chain and the α-chain. As predicted by modeling and assessed by SPR binding assays, we found the following progression in *K*_D_ (Table 1): WT (21.4 μM) > β-G50A (4.6 μM) > β- G50A + A51E (1.9 μM) > β-G50A + A51E + A97L (0.91 μM) > β-G50A + βA51E-βA97L + α-S53W (0.14 μM) (Zoete et al., in preparation) (7). Our method thus allows for the design of TCRs with fine tuned *K*_D_ values, potentially leading to T cells with optimal activity (7). This is in contrast with experimental display approaches which tend to select the tightest-binding TCRs that can potentially surpass an “affinity threshold of specificity,” as has been reported for the 1G4 TCR (107). We found a very satisfying correlation between the calculated energies and the p*K*_D_ and *k*_off_ values measured by SPR (Zoete et al., in preparation).

**Table 1:** **Binding affinity of rationally designed BC1 TCR variants [from Ref. (7)] measured by SPR**.

TCR mutations	*K*_D_ (μM)
β-V49I	Near non-binding
Wild-type	21.4
β-G50A	4.62
β-A97L	2.69
β-G50A + A51E	1.91
β-G50A + A51E + A97L	0.91
α-S53W + β-G50A + A51E	0.4
α-S53W + β-G50A + A51E + A97L	0.14
β-G50A + A51I + G52Q + I53T	0.015

**Figure 11:**
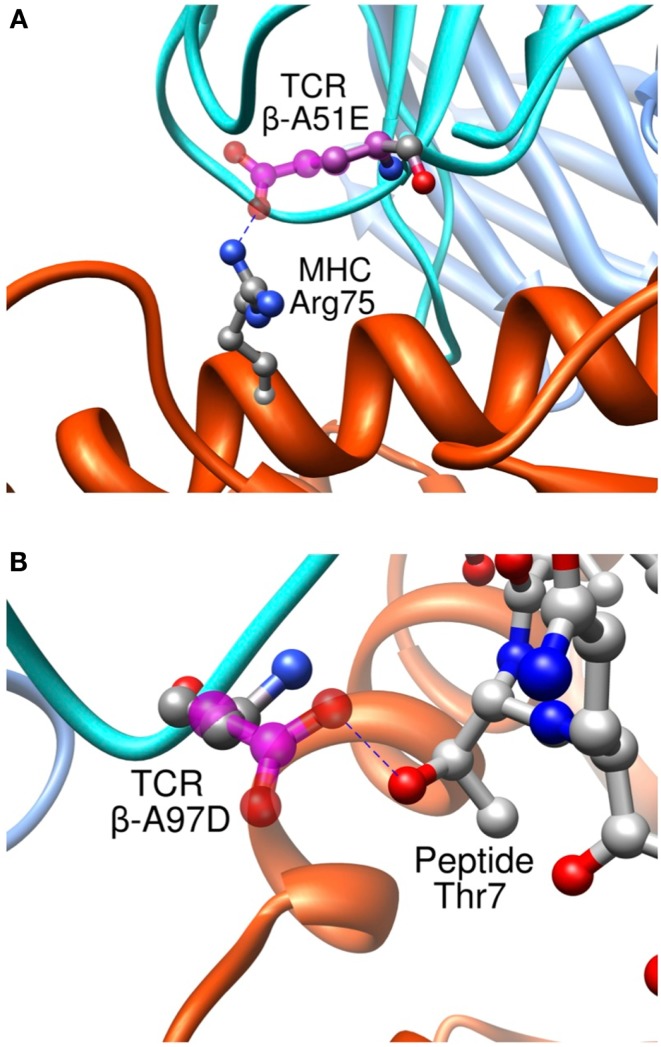
**Interaction between pMHC and the modified residues of the β-A51E (A) and β-A97D TCR variants (B)**. TCR wild-type residues are shown in ball and stick, colored according to the atom types. Modified residues are colored in magenta. New hydrogen bonds are shown as dotted blue lines.

Importantly, we found that our approach was able to predict successfully mutations toward both non-polar and polar residues, contrary to previous studies where only designed mutations toward non-polar residues were successful in increasing the experimental affinity (116). To take place, polar interactions, such as hydrogen bond or ionic interaction, require an appropriate match of chemical functionalities and precise geometrical constrains between interacting partners. Therefore, they provide an essential contribution to the directionality and the specificity of molecular recognition (127). This point is critical to the development of TCRs for immunotherapies. It is indeed essential that TCRs maintain their specificity, and do not acquire novel antigen specificities that might cause off-target toxicity upon the adoptive transfer of genetically engineered T cells to patients. Two rationally designed sequence modifications provide interesting examples regarding the detailed control provided by our approach on the binding process. Mutation β-A51E introduces an ionic interaction between the new glutamate side-chain and MHC Arg75 (Figure 11A), translating into a calculated favorable ΔΔ*G*_bind_. This replacement produced a fourfold experimental improvement in *K*_D_ as measured by SPR (8). This mutation thus increases the affinity for the pMHC through better interactions with the MHC molecule, and therefore cannot be expected to increase the selectivity for the peptide antigen. Interestingly, we also designed the β-A97D variant, which introduces a new hydrogen bond between TCR and the peptide Thr7 side-chain (Figure 11B). The overall effect of this modification was somewhat unfavorable to the binding, as shown by the experimental titration ELISA and calculated ΔΔ*G*_bind_ values (Zoete et al., in preparation). However, despite the decreased activity, this mutation is interesting for the putative gain in selectivity for the NY-ESO-1 system thanks to the new polar interaction taking place between the TCR and the peptide.

An experimental alanine scanning of the peptide supported this idea. The affinity of several TCR mutants (β-G50A/A51E, A97L, A97D, I53W, V95L, and Y94N) for the different mutated pMHC were measured by titration ELISA and compared to the results for the wild-type TCR. All mutated TCR showed affinities similar to the wild-type TCR for all the peptide mutants. The only exception was the β-A97D TCR mutant, which did not bind to the peptide T7A mutant contrary to the wild-type TCR. This experimental result is in good agreement with the *in silico* data showing that all those TCR mutations modified the interactions with the MHC rather than with the peptide with the exception of β-A97D which exchanges a hydrogen bond with the peptide Thr7 side-chain. The β-A97L TCR mutant, which makes non-polar contact with the side-chain of peptide Thr7 but no hydrogen bond, is less affected by the Thr7 mutation to Ala than the β-A97D TCR variant. Thus, although the β-A97D modification is somewhat unfavorable to the binding, it could be useful to introduce it in engineered TCRs targeting NY-ESO-1, in addition to other variations increasing the affinity, in order to obtain more selective TCRs.

The selectivity of TCRs designed by our approach was tested experimentally. We observed no interaction of our TCRs with any non-cognate pMHC complexes. In addition, tetramer binding studies with an extensive panel of non-cognate pMHC revealed that the TCR variants expressed at the cell-surface were also HLA-A^∗^0201/NY-ESO-1_157–165_ –specific (7).

The binding free energy calculations used in our approach are physics-based and not reliant on *ad hoc* model fitting. We can thus expect that our design strategy is highly transferable to any protein/protein interaction of known structure and biological interest.

### Intermediate TCR/pMHC binding parameters confer maximum biological responses

In order to efficiently screen our extensive panel of modeled BC1 TCR variants we began by establishing a mammalian cell expression system for TCR production. HEK-293 cells were PEI co-transfected with pHYK8 plasmids encoding the α- and β-chains, each under control of the CMV promoter. Following the strategy of Chang et al. an acidic-basic zipper was incorporated to facilitate heterodimeric chain pairing (128). Following 5–7 days culture in serum-free medium, the TCR variants were HIS-tag purified from the supernatants (yields were up to ∼3 mg/L) and compared to the wild-type TCR by titration ELISA for binding plate-captured pMHC. Single and multiple amino acid replacements were assessed in the α- and β-chains. Over 60% showed enhanced pMHC binding as compared to the wild-type TCR. Further, binding of the TCR variants against an Ala replacement scan of the NY-ESO-1_157–165_ peptide (SLLMWITQC) revealed near identical patterns of recognition, suggesting a conserved mechanism of binding. For all TCR variants, M160, W161, I162, and Q164 were critical contact residues as their replacement with Ala abolished TCR/pMHC binding (7, 8).

The TCR chains were subcloned into pGMT7 for their expression as inclusion bodies in BL21(DE3)pLys bacterial cells and subsequent TCR refolding by dialysis (129). The binding affinity and kinetics for a panel of TCRs having various combinations of amino acid replacements in CDR2-β, CDR3-β, and CDR2-α, were measured by SPR using the BIACore 3000. Most natural TCRs bind pMHC with weak affinity, in the range of 1–100 μM, as a result of slow association (10^3^–10^4^ M^−1^s^−1^), and fast rates of dissociation (typically a half-life of seconds at 37°C) (1, 10, 130), reflecting the need for T lymphocytes to detect a virtually limitless repertoire of foreign epitopes while avoiding autoreactivity, and the fact that they do not undergo somatic hypermutation as do antibodies. The eight TCRs chosen amongst our panel incrementally increased in affinity from 21.4 μM for the wild-type one, to extreme physiologic affinity at 15 nM (summarized in Table 1).

**Figure 12:**
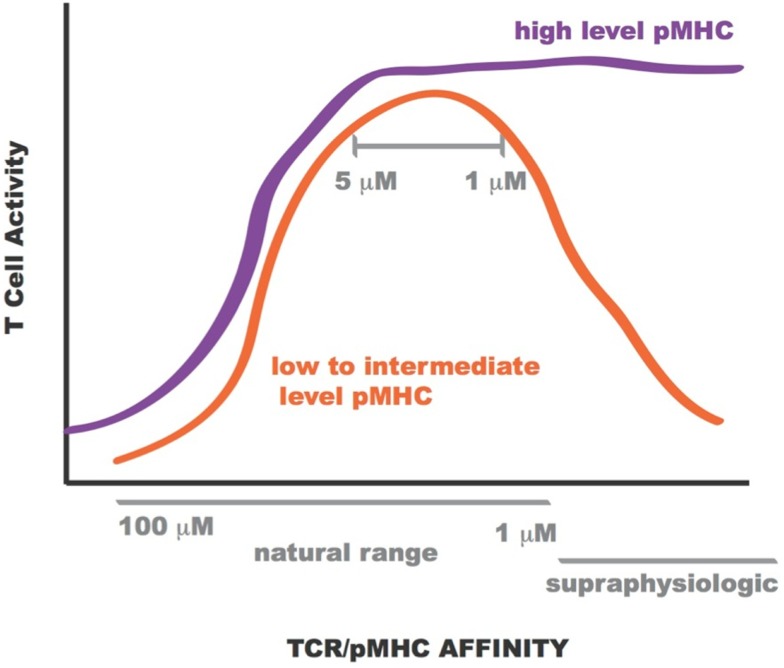
**Sketch of T cell activity as a function of TCR-pMHC affinity**. At low to intermediate levels of pMHC, T-cell activity is optimal for TCR within *K*_D_ range 1–5 μM (orange curve). At high levels of pMHC T-cell activity is not attenuated for supraphysiologic affinity TCR (purple curve).

Lentiviral constructs were built to assess the activity of the rationally designed TCRs in transduced primary human CD8^+^ T cells and identify those able to confer maximum activity levels. A range of functions were assessed for the transduced CD8^+^ T cells including Ca^2+^ flux, intracellular signaling, cell-surface TCR clustering, target-cell killing, and cytokine/chemokine release. All activity levels for affinity-enhanced TCRs uniformly increased from that of the wild-type TCR, reaching a peak for TCRs within the upper range of natural affinity, 1–5 μM. Beyond this affinity, in the supraphysiological range, however, activity levels began to drop, both in the presence and absence of CD8 co-receptor engagement, usually reaching a minimum for the extreme supraphysiological affinity TCR (1, 7, 8, 10, 130). However, under experimental conditions in which the T cells were exposed to target-cells pulsed with high concentration of NY-ESO-1_157–165_ peptide this attenuation in activity for the supraphysiological affinity TCR was no longer observed. This phenomenon is illustrated in Figure 12. Importantly, no non-specific reactivity was observed for any of the rationally designed TCRs (7, 8).

**Figure 13:**
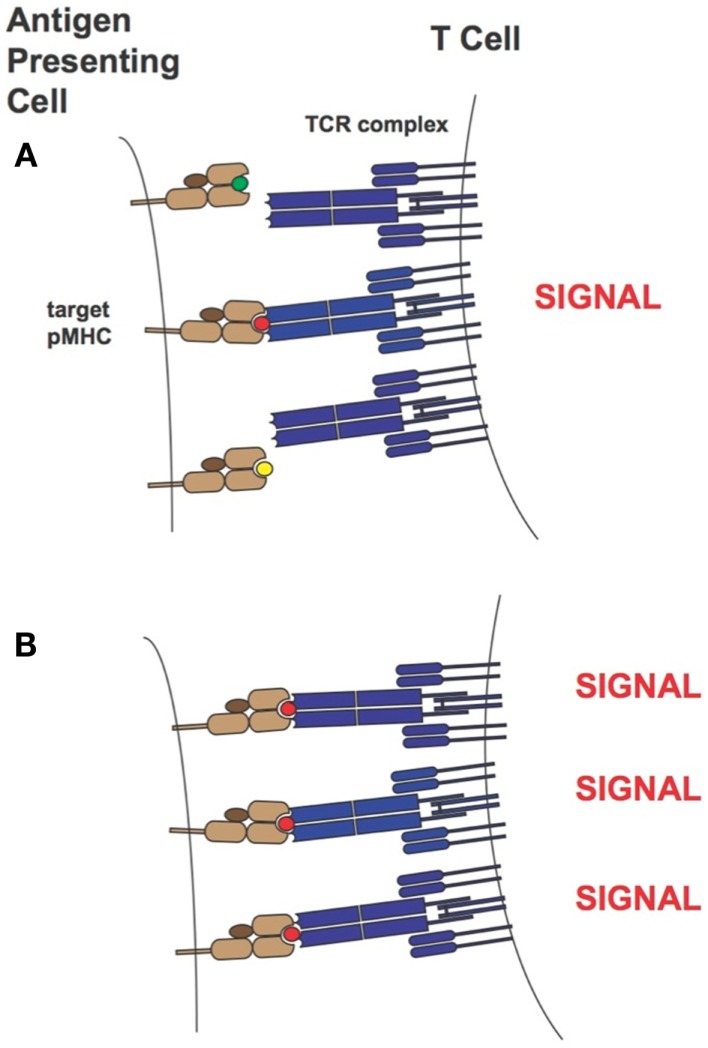
**T-cell activation in the presence of limiting (A) or saturating levels (B) of target pMHC complex**. In the case where target pMHC concentration is limiting, TCRs of too high-affinity may inhibit serial engagement and thereby attenuate T-cell activity levels. MHCs are colored in light brown, β2-microglobulins in dark brown, TCRs in blue and peptide antigens are shown as small green, red, and yellow circles. The peptide colored in red corresponds to the specific antigen.

Over the years two main models of T cell activation have emerged [reviewed in Stone et al. (11)]. For the “affinity model” (101, 131) the total number of TCRs bound to pMHCs at equilibrium is thought to regulate T cell activity levels. The “half-life model” proposes that the TCR must stay bound with sufficiently strength/duration for productive signaling and also enable the serial engagement of the “rare” antigenic pMHC complex by adjacent TCR for the amplification of signal (i.e., the TCRs must have an optimal dwell-time) (96, 97, 132–134). Although the half-life of a TCR/pMHC interaction has traditionally been calculated from *k*_off_ values (*t*_1/2_ = ln2/*k*_off_), recent work by Aleksic et al. has elegantly demonstrated that for TCRs having faster association rates there can be rapid TCR/pMHC re-engagement rather than a lateral diffusion of TCRs in the cell membrane to prolong the effective half-life of the TCR/pMHC interaction. Thus, both *k*_off_ and *k*_on_ can contribute to TCR/pMHC dwell or confinement time (135). Overall our findings correspond to the “half-life” model of T cell activation. Presumably the supraphysiological affinity TCRs are limiting the serial engagement but at high peptide concentrations this is not an issue as the TCR can find more pMHC molecules on the target-cell-surface. This is demonstrated in Figure 13. Our work also demonstrated the value of a modeling approach because TCRs with particular binding parameters, i.e., falling within an optimal affinity range, either through faster on-rates or slower off-rates, can be developed to enable maximum T cell activity levels.

## Conclusion

We found that the synergy between *in silico* design, and *in vitro* testing using both soluble molecules and transfected cells is key to the design of improved TCRs to be used in adoptive cell transfer therapies. Using an innovative structural approach based on recent *in silico* techniques, we have developed a method to rationally optimize the surface of the TCR to increase its affinity for pMHC. As opposed to library screening, we propose a step-wise, incremental optimization of the TCR. This allows the preservation of the favorable initial properties of the TCR through the various cycles. Our optimization method can selectively target contacts involving the MHC or the peptide, thus maintaining a good balance between overall affinity and specificity. In addition, modifying only a few amino acids reduces the risk of obtaining undesired cross-recognition or raising anti-TCR antibodies *in vivo*. This rational optimization approach is therefore very promising to the design of TCRs for adoptive T cell immunotherapy clinical trials.

The application of our TCR engineering approach to the tumor-targeting BC1 TCR targeted to the A2/NY-ESO-1_157–165_ antigen identified several original single mutations of the CDR loops conferring increased experimental affinity for pMHC compared to the wild-type TCR. T cells expressing some of the affinity-enhanced TCR showed better overall functionality, including improved killing of both peptide-loaded T2 cells and melanoma tumor cell lines, higher proliferative capacity, and increased levels of cytokine/chemokine secretion, as compared to wild-type TCR expressing T cells. For all functional assays, we observed a gain in CD8^+^ T cell activity level with increase in affinity, with a peak at an affinity of ∼1–5 μM. Beyond this affinity we observed a progressive decrease in activity levels. We are currently testing the relative activity of the different TCRs *in vivo* in a mouse model. The successful candidates are planned to enter a phase I clinical trial program for stage IV melanoma. As the methods presented here are general and transferable to any TCR-pMHC complex, other cancer types will follow shortly.

In parallel to direct applications of the existing approach, we will take advantage of the methodological work presented above to improve our *in silico* TCR optimization method. The development of homology-based approaches to model the 3D-structure of TCR-pMHC complexes potentially extends the use of our method to TCR repertoires for which no X-ray structure is available. The detailed picture of the TCR/pMHC interaction emerging from the MD simulations showed the presence of single water molecules trapped at the interface (20). Including these interfacial waters in the BFED scheme would improve accuracy in some cases. Close residues at protein–protein interfaces can display collaborative effects that result in the non-additivity of their contributions to the binding free energy, which will have to be taken into account in the next generation of TCR optimization methods. Noticeably, because the binding free energy decomposition method used is physics-based, without any *ad hoc* parameters, our *in silico* techniques are straightforwardly transferable to other types of macromolecular protein complexes.

## Conflict of Interest Statement

The authors declare that the research was conducted in the absence of any commercial or financial relationships that could be construed as a potential conflict of interest.
